# Expanded-spectrum β-Lactamase and Plasmid-mediated Quinolone Resistance

**DOI:** 10.3201/eid1305.061293

**Published:** 2007-05

**Authors:** Laurent Poirel, Laura Villa, Alessia Bertini, Johann D. Pitout, Patrice Nordmann, Alessandra Carattoli

**Affiliations:** * Hôpital de Bicêtre, K.-Bicêtre, France; † University of Calgary, Calgary, Alberta, Canada; ‡ Istituto Superiore di Sanita, Rome, Italy

**Keywords:** quinolone resistance, extended-spectrum β-lactamase, QnrA, VEB-1, plasmid, letter

**To the Editor:** The emergence of plasmid-mediated, and thus transferable, quinolone resistance determinants has been recently discovered (*1*) and shown to involve the pentapeptide repeat protein Qnr, which interacts with DNA gyrase and topoisomerase IV to prevent quinolone inhibition (*2*,*3*). Qnr determinants confer resistance to nalidixic acid and reduced susceptibility to fluoroquinolones (*3*). They have been identified worldwide in a variety of enterobacterial species and were often associated to expanded-spectrum β-lactamases (ESBLs) (*2*). The association between the ESBL VEB-1 and the QnrA1 determinants was reported (*4*). Because plasmid co-localization of QnrA and VEB-1 encoding genes has been reported repeatedly from scattered clonally-unrelated enterobacterial isolates, our objective was to use replicon typing to trace a possible dissemination of a common plasmid worldwide.

The *bla*_VEB-1_ and/or *qnrA*-positive plasmids that have been included in the study were from 17 isolates previously described in detail (*3*–*8*) (Table). *Escherichia coli* transconjugants (Tc) were obtained for 14 of 17 clinical isolates, allowing an accurate replicon typing since original clinical isolates might harbor several plasmids. They were collected from 1999 to 2005, from patients hospitalized in different parts of the world (Table). The 13 *bla*_VEB-1_-positive isolates were from 5 countries (France, Turkey, Algeria, Thailand, and Canada), scattered on 4 continents. Among them, the *Providencia stuartii* and *Proteus mirabilis* isolates from Algeria were negative for *qnrA1*. In addition, 4 *bla*_VEB-1_-negative but *qnrA1*-positive isolates recovered from France and Australia were also included in the study.

**Table:** Features of the VEB-1– or QnrA-positive isolates used in this study*

Strain†/Ref.	Spp. of origin	Country	Year of isolation	Plasmid size (kb)	ESBL	QnrA1	Replicon	Associated non–β-lactam resistance markers
Escherichia coli TcE1 (*5*)	*E. coli*	Thailand	1999	160	VEB-1	+	A/C_2_	NAL, K, SSS, C, RA
E. coli TcE4 (*5*)	*E. coli*	Thailand	1999	150	VEB-1	+	A/C_2_	NAL, K, TM, SSS, C, RA
E. coli TcE5 (*5*)	*E. coli*	Thailand	1999	150	VEB-1	+	A/C_2_	NAL, K, TM, SSS, SXT
E. coli TcE7 (*5*)	*E. coli*	Thailand	1999	150	VEB-1	+	A/C_2_	NAL, K, TM, SSS, C, RA
E. coli TcE8 (*5*)	*E. coli*	Thailand	1999	150	VEB-1	+	A/C_2_	NAL, K, TM, SSS, TE
*E. coli* TcE16 (*5*)	*E. coli*	Thailand	1999	140	VEB-1	+	A/C_2_	NAL, K, TM, SSS, RA
*E. coli* TcE18 (*5*)	*E. coli*	Thailand	1999	180	VEB-1	+	A/C_2_	NAL, K, SSS, C, RA
*E. coli* Tc(pl) (*5*)	*E. coli*	Canada	2000	180	VEB-1	+	A/C_2_	NAL, K, SSS, C, RA
*E. coli* Tc(pQR1) (*4*)	*E. coli*	France	2003	180	VEB-1	+	A/C_2_	NAL, K, SSS, C, RA, SXT
*E. coli* Tc(GOC) (*4*)	*Enterobacter cloacae*	France	2003	190	VEB-1	+	A/C_2,_ FIB	NAL, K, TM, SSS, C
*Citrobacter freundii* LUT (3)	*C. freundii*	Turkey	2004	ND	VEB-1	+	A/C_2,_ FIB, K	NA
*Providencia stuartii* 15 (this study)	*P. stuartii*	Algeria	2004	ND	VEB-1	–	A/C_2_	NA
*E. coli* TcMAA (this study)	*Proteus mirabilis*	Algeria	2004	190	VEB-1	–	A/C_2_	K, TM, SSS, C, SXT
*E. coli* TcK147 (*7*)	*Klebsiella pneumoniae*	Australia	2002	160	SHV-12	+	HI2, A/C_1_, P	NAL, K, TM, C, TE, SXT
*E. cloacae* A1 (*8*)	*E. cloacae*	France	2004	75	SHV-12	+	HI2	NA
E. coli TcA2 (*8*)	*Enterobacter aerogenes*	France	2005	150	SHV-12	+	FII	NAL, K, TM, TE
E. coli TcA3 (*8*)	*K. pneumoniae*	France	2005	40	–	+	I1, K	NAL, K, TM, C, TE

*Ref., reference; ESBL, extended-spectrum β-lactamase; NAL, nalidixic acid; K, kanamycin; SSS, sulfonamides; C, chloramphenicol; RA, rifampin; TM, tobramycin; SXT, trimethoprim-sulfamethoxazole; TE, tetracycline; ND, not determinable; NA, not applicable. †Tc indicates that this is a transconjugant or a transformant.

PCR-based replicon typing (PBRT), which recognizes FIA, FIB, FIC, HI1, HI2, I1-Ig, L/M, N, P, W, T, A/C, K, B/O, X, Y, and FII replicons (*9*), was applied to type the resistance plasmids from all the strains. Amplicons were confirmed by DNA sequencing and used as probes in hybridization experiments on purified plasmids (data not shown).

PBRT results showed that the 13 *bla*_VEB-1_-positive plasmids (including 11 *qnrA1*-positive) belonged to the IncA/C incompatibility group. DNA sequencing identified the A/C_2_ replicon variant (European Molecular Biology Laboratory no. AM087198) in all these plasmids (Table). Plasmids of this type were recently identified in the United States and in Italy carrying the AmpC-type cephalosporinase CMY-4–encoding gene (*10*). In 2 strains (*E. coli* TcGOC and *Citrobacter freundii* LUT), the IncA/C_2_ plasmids were associated with additional replicons, which suggests the presence of multiple plasmids or fusions between plasmids of different backbones. By contrast, all the 4 *bla*_VEB-1_-negative isolates but *qnrA1*-positive were negative for the A/C replicon, except transconjugant TcK147; however, sequencing identified an A/C_1_-type replicon in that strain. These results indicated that the genes encoding QnrA1 and VEB-1, when identified concomitantly in a given isolate, were always located on plasmids belonging to the same IncA/C_2_-incompatibility group that may vary in size and digestion pattern (Table; unpub. data). In addition, we showed that plasmids carrying the *bla*_VEB-1_ gene but lacking *qnrA1* were also of the IncA/C_2_ type (Table). Plasmids that were *bla*_VEB-1_-negative but *qnrA1*-positive were of distinct replicon types, thus suggesting independent acquisition of the *qnrA1* gene on different plasmids. It is remarkable that since VEB-1 is apparently always encoded by IncA/C_2_ plasmids, when genes for QnrA1 and VEB-1 are found together, they also occur on IncA/C_2_ plasmids.

Thus, evidence here shows that the IncA/C_2_ plasmid is the main vehicle of the *bla*_VEB-1_ gene worldwide, on which the *qnrA1* gene may be added. The possibility that both *bla*_VEB-1_ and *qnrA1* genes may be identified on a single genetic structure in several isolates has been recently shown with their identification within the same *sul1*-type integron (*6*).

Since results of these experiments provided a good marker for tracing *bla*_VEB-1_-positive plasmids, and taking in account the property of A/C-type plasmids to have a broad range of hosts (note: this has not been demonstrated for the specific A/C_2_ subgroup), we tried to amplify the A/C_2_ replicon in a collection of 15 *bla*_VEB-1_-positive and clonally unrelated *Pseudomonas aeruginosa* isolates from France, Thailand, India, and Kuwait. The *bla*_VEB-1_ gene was supposed to be chromosome-encoded in those isolates. PCR failed to give any positive results, confirming the absence of an IncA/C-type plasmid and also ruling out the hypothesis of IncA/C_2_-type plasmid co-integration at the origin of *bla*_VEB-1_ acquisition in *P. aeruginosa*.

The spread of plasmids carrying a large array of resistance genes among *Enterobacteriaceae* is of concern since this provides a convenient genetic mechanism for a given strain to become panresistant to antimicrobial drugs. In particular, the recent identification of the Qnr determinants have shown that plasmids may provide resistance (or at least reduced susceptibility) to quinolones and fluoroquinolones, whereas they are already known to carry resistance to β-lactams, aminoglycosides, chloramphenicol, tetracycline, rifampin, sulfonamides, and disinfectants. pQR1 (*4*) or p1 (*6*) are examples of well-characterized plasmids that mediate multidrug resistance by carrying *bla*_VEB-1_ and *qnrA1*, together with aminoglycoside resistance genes *aadB*, *aacA1*, and *aadA1*, chloramphenicol resistance gene *cmlA*, rifampin resistance gene *arr2*, disinfectant resistance gene *qacI*, and sulfonamides resistance gene *sul1*.

Our study showed that the IncA/C_2_-type plasmids may be the source of such worldwide dissemination. It means that 1 plasmid scaffold has brought the same (or at least very similar) multidrug resistance to multiple enterobacterial species in different continents.
